# Aphids transform and detoxify the mycotoxin deoxynivalenol via a type II biotransformation mechanism yet unknown in animals

**DOI:** 10.1038/srep38640

**Published:** 2016-12-08

**Authors:** N. De Zutter, K. Audenaert, N. Arroyo-Manzanares, M. De Boevre, C. Van Poucke, S. De Saeger, G. Haesaert, G. Smagghe

**Affiliations:** 1 Department of Crop Protection, Laboratory of Agrozoology, Faculty of Bioscience Engineering, Ghent University, Coupure Links 653, 9000 Ghent, Belgium; 2 Department of Applied Biosciences, Faculty of Bioscience Engineering, Ghent University, Valentin Vaerwyckweg 1, 9000 Ghent, Belgium; 3 Department of Bioanalysis, Laboratory of Food Analysis, Ghent University, Ottergemsesteenweg 460, 9000 Ghent, Belgium

## Abstract

Biotransformation of mycotoxins in animals comprises phase I and phase II metabolisation reactions. For the trichothecene deoxynivalenol (DON), several phase II biotransformation reactions have been described resulting in DON-glutathiones, DON-glucuronides and DON-sulfates made by glutathione-S-transferases, uridine-diphosphoglucuronyl transferases and sulfotransferases, respectively. These metabolites can be easily excreted and are less toxic than their free compounds. Here, we demonstrate for the first time in the animal kingdom the conversion of DON to DON-3-glucoside (DON-3G) via a model system with plant pathogenic aphids. This phase II biotransformation mechanism has only been reported in plants. As the DON-3G metabolite was less toxic for aphids than DON, this conversion is considered a detoxification reaction. Remarkably, English grain aphids (*Sitobion avenae*) which co-occur with the DON producer *Fusarium graminearum* on wheat during the development of fusarium symptoms, tolerate DON much better and convert DON to DON-3G more efficiently than pea aphids (*Acyrthosiphon pisum*), the latter being known to feed on legumes which are no host for *F. graminearum.* Using a non-targeted high resolution mass spectrometric approach, we detected DON-diglucosides in aphids probably as a result of sequential glucosylation reactions. Data are discussed in the light of an eventual co-evolutionary adaptation of *S. avenae* to DON.

Fusarium Head Blight (FHB) is an important disease on small grain cereals such as wheat, barley and oats. Although the disease is caused by a species complex, *F. graminearum* is considered as the most important species in the complex^1^. The risk associated with infection by this species comes primarily from the toxic secondary metabolites, mycotoxins, that *F. graminearum* deposits in the host matrix.

Trichothecenes are a class of mycotoxins produced by several fungal species of the genus *Fusarium* and related genera in agricultural crops. They belong to the structural group of sesquiterpenoids, all bearing a common tricyclic 12, 13-epoxytrichothec-9-ene core structure. Type A, B, C and D trichothecenes can be distinguished based on substitutions at position C-4, C-7, C-8 and/or C-15^1^. Worldwide the type B trichothecene deoxynivalenol (DON, Fig. 1) is one of the most important mycotoxins because of its omnipresence in many cereal-based matrices^1^,^2^,^3^. DON provokes acute and chronic disease symptoms in humans and animals. Its toxic effects range from diarrhea, vomiting, gastro-intestinal inflammation, necrosis and apoptosis of the intestinal tract, the bone marrow and the lymphoid tissues^4^,^5^. DON causes inhibition of the mitochondrial function and has effects on cell division and membrane integrity. Finally, it also inhibits protein, DNA- and RNA synthesis in eukaryotic cells^6^,^7^,^8^. The toxicity of these sesquiterpenes can be explained by their chemical structure containing an epoxide at the C-12 and C-13 position (Fig. 1)^9^. Although most eukaryotic organisms are susceptible to trichothecenes to a certain level, many of them have developed strategies to arm themselves against the detrimental effects of these mycotoxins, and examples are present throughout the fungal, animal and plant kingdom.

In the yeast *Saccharomyces cerevisiae,* a spontaneous mutant tolerant to the trichothecene, trichodermin was isolated^7^. The tolerance was shown to be based on alteration of the target side of trichothecenes. The gene responsible for the trichodermin tolerance was called *tcm1*^8^ and was suggested to encode for the ribosomal protein L3 (RPL3)^10^, which is the target of trichothecenes. The DNA sequence of *tcm1* was determined^11^ and a mutation in this gene did not only cause tolerance to trichothecenes, but also affected the maturation of either 40 S or 60 S ribosomal subunits^12^. Similarly, Mitterbauer *et al*.^13^ depicted several mutations in *Rpl3* conferring semi-dominant resistance to trichothecenes^13^. Transgenic tobacco plants expressing a modified *Rpl3* cDNA were shown to be able to adapt to DON. However, the tolerance was not consistent because the engineered RPL3 protein which shows a lower affinity to the ribosome assembly factor Rrb1p, was not utilized by the translational machinery in the presence of the native RPL3^13^.

In animals, two major metabolic pathways for detoxification of trichothecenes have been reported. A well-known example is the deepoxidation of the trichothecene DON to deepoxidated DON (DOM-1) in cows and pigs amongst others^14^. In addition, several type II biotransformation reactions have been reported in which DON is conjugated with glucuronides, sulphonates or glutathione^15^. In contrast to the vast amount of data on mammals, information on transformation and detoxification strategies in insects remains limited. Nevertheless, insects often live in close proximity of trichothecene-producing fungi and the toxicity has been reported in a few studies. Trichodermin and other 12,13-epoxytrichothecenes have been shown to have larvicidal activity against mosquitoes of *Aedes aegypti*^16^. DON has toxic effects on cells of the lepidopteran *Spodoptera frugiperda*^17^. In addition, the trichothecenes (type A) diacetoxyscirpenol and neosolaniol are demonstrated to be potent anti-feedants against *Galleria* mellonella larvae^18^.

Trichothecenes are also prone to metabolization in plants. The major detoxification process of trichothecenes in plants is through the action of glucosyltransferases which insert a glucose rendering the toxin more water soluble and redirecting it to the vacuoles^19^. Furthermore, several studies have reported the detection of conjugation products of DON with glutathione (DON-GSH) and the degradation products DON-S-cysteinylglycine (DON-S-cys-gly) and DON-S-cysteine (DON-S-cys)^20^,^21^,^22^.

Finding new detoxification strategies for mycotoxins is a growing field of interest and the first step in order to implement this knowledge in future mycotoxin remediation strategies. New insights on detoxification might come from animals that live in close contact with DON-producing fungi. A well-known example is the English grain aphid (*Sitobion avenae*) which migrates from the leaves to the developing ear upon its appearance. From that moment, it resides on the same niche as the *Fusarium* fungus and comes in close contact with its toxic secondary metabolites. The present study aimed to assess the ability of these plant-pathogenic aphids to cope with the trichothecene DON. We demonstrated the presence of type II biotransformation mechanisms linked with DON detoxification in two aphid species. We used a non-targeted high resolution mass spectrometric approach to get an in depth insight into eventual new derived metabolites of DON in animals.

## Results

### Survival of *S. avenae* and *A. pisum* upon exposure to DON

*S. avenae* and *A. pisum* aphids were fed for 3 days on a diet containing different concentrations of DON (0, 0.5, 1, and 3 mg l^−1^) using an aphid feeding apparatus. These concentrations were chosen because they are relevant physiological concentrations in the field: from surveys, it is known that DON levels range from 0 till 5 mg kg^−1^ at field level (unpublished data). In addition, at the level of a spikelet, these concentrations can mount to 50 mg kg^−1^ (unpublished data).The survival of *S. avenae* aphids was not affected by DON up to concentrations of 3 mg l^−1^ compared to the control when analyzed using a Kruskal-Wallis test (p = 0.337), while survival of *A. pisum* aphids was significantly reduced. The lowest administered concentration of 0.5 mg l^−1^ DON significantly reduced (p = 0.024) the survival rate of *A. pisum* (62% ± 6) compared to the control treatment which was 100% (Table 1). As we wanted to assess the tolerance of *S. avenae* in more detail, we exposed both aphid species to a concentration of 100 mg l^−1^ DON for 3 days. Remarkably 43% ± 8 of the *S. avenae* aphids survived this dose, while for the *A. pisum* survival rates dropped to 4% ± 2. The LC 50 values of DON for *S. avenae* and *A. pisum* were 62.9 mg l^−1^ and 2.43 mg l^−1^ respectively.

### Involvement of RPL3 in the tolerance of *S. avenae* to DON

Previous research has reported increased tolerance to DON by amino acid modifications in the RPL3 protein which is the target of DON^13^. For that reason, we sequenced the nucleotide sequence of the gene encoding for RPL3 of *S. avenae* and *A. pisum*. After converting the nucleotide sequence to amino acids, no differences were found between the RPL3 sequence of *S. avenae* when aligned with 60 S RPL3 of *A. pisum* (NCBI Reference Sequence: XP_001951042.1) and with other insects (Supplementary Figure 1). The typical amino acid changes observed in *Saccharomyces cerevisiae* which were associated with DON tolerance were not detected in any of the aphid species.

**Figure 2:**
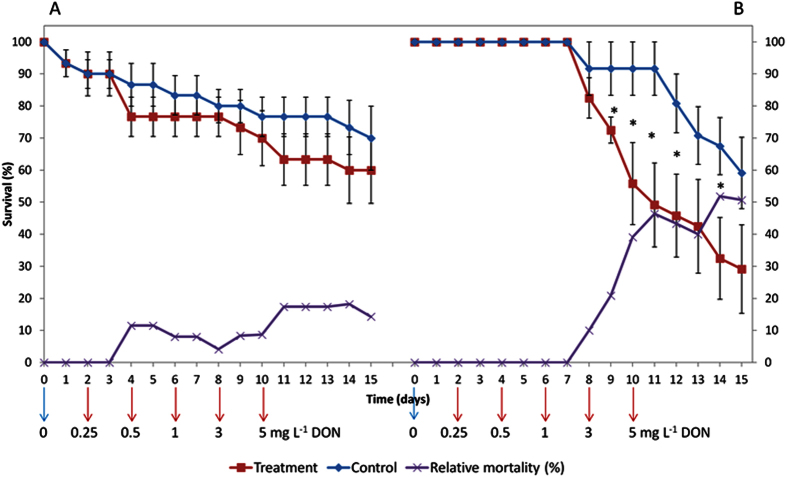
Long-term survival of *S. avenae* aphids (**A**) and *A. pisum* aphids (**B**) (means ± SE) feeding on diet with increasing concentrations of deoxynivalenol (DON) (0 → 5 mg l^−1^). The purple line indicates the mortality of the aphids relative to the surviving fraction at each time point. *Indicates significant differences (p < 0.05) between treatment and control using one-sided t-tests.

Experiments with transformed plants, have shown that an eventual modified DON insensitive RPL3 protein can be present heterozygously^13^. In this case, the insensitive RPL3 protein is not used by the translation machinery in the presence of the native RPL3 protein due to a lower affinity of the mutant RPL3 for the ribosome assembly factor Rrb1p. In this scenario, the mutant RPL3 protein only accumulates when organisms are gradually exposed to DON which allows the mutant RPL3 to push out the native RPL3 protein. In order to investigate whether a similar adaptation mechanism was present in *A. pisum,* aphids were exposed to an increasing concentration of DON in a time-lapse experiment. However, feeding *A. pisum* with increasing concentrations of DON during a longer period of time did not result in an increased survival (Fig. 2). As expected, the survival of *S. avenae* was not negatively influenced by increasing DON concentrations.

### Conversion of DON to DON-3G in aphids results in a detoxification

In order to get an insight into the ability of the aphids to detoxify DON, aphids were fed 100 mg l^−1^ of DON in an aphid feeding apparatus. In the control aphids which fed on artificial diet only, no DON or DON derivatives were detected. In addition, we tested eventual chemical- or photo-degradation of DON in the artificial diet not exposed to aphids. However, in the artificial diet amended with 100 mg l^−1^ DON, we found 99.37 ± 0.83 mg l^−1^ DON illustrating that DON was not degraded in the feeding apparatus during the course of the experiment. Using an enzyme based approach we could not demonstrate an involvement of glutathione-S-transferases (GSTs) or cytochrome P450 monooxygenases (P450s) in the detoxification process (data not shown).

In search for another explanation, a targeted liquid chromatography coupled to tandem mass spectrometry (LC-MS/MS) approach was used. Aphids which were exposed to DON were analyzed for DON (LOD, 45 μg kg^−1^; LOQ, 89 μg kg^−1^), DON-3G (LOD, 34 μg kg^−1^; LOQ, 67 μg kg^−1^) 3- and 15-acetyldeoxynivalenol (3-ADON: LOD, 47 μg kg^−1^; LOQ, 94 μg kg^−1^) and 15-ADON (LOD, 33 μg kg^−1^; LOQ, 67 μg kg^−1^). For structures see Supplementary Figure 2.

**Figure 3:**
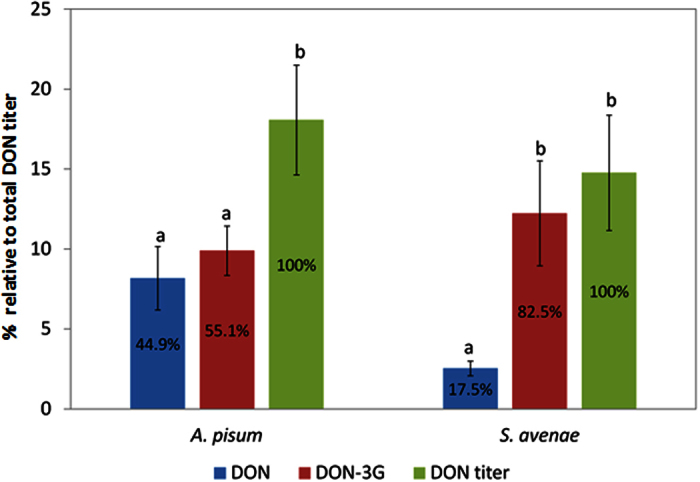
Relative concentrations (%) of deoxynivalenol (DON), deoxynivalenol-3-glucoside (DON-3G) compared to the total DON titer in *S. avenae* and *A. pisum* aphids after two days of feeding on artificial diet amended with 100 mg l^−1^ DON. Different letters indicate significant differences (p < 0.05) between treatments using a one-way ANOVA with a post-hoc Tukey test.

Remarkably, DON-3G, a type II-conjugate of DON that is solely reported in plant detoxification pathways, was detected. Moreover, there was a clear difference in the metabolisation efficiency between both aphid species. For DON-3G *A. pisum* contained 9.9 ± 1.6 mg/kg while in *S. avenae* 12.5 ± 3.0 mg/kg DON-3G was present. Remarkably, the DON levels in both aphid species differed significantly (p = 0.017): In *A. pisum* 9.0 ± 1.5 mg/kg DON was recovered while in *S. avenae* only 2.5 ± 0.4 mg/kg was detected. This result points to a more efficient conversion of DON to DON-3G in *S. avenae*. This was even more clear when we considered relative amounts of DON. *S. avenae* aphids (tolerant to DON) efficiently converted DON to DON-3G (82.5% of the total DON titer was present as DON-3G) and both were significantly different (p = 0.000), whereas in *A. pisum* only 55% of the total DON titer consisted of DON-3G and DON-3G levels did not differ significantly from DON levels (p = 0.812) (Fig. 3). In view of these results, the toxicity of DON-3G in aphids was assessed by exposing both *S. avenae* and *A. pisum* to DON-3G concentrations of 0.5 mg l^−1^, 1 mg l^−1^, and 3 mg l^−1^. These experiments clearly demonstrated that DON-3G was no longer toxic for either aphid species (Table 1).

### DON-3G can be further metabolized: diglucosides of DON

**Figure 4:**
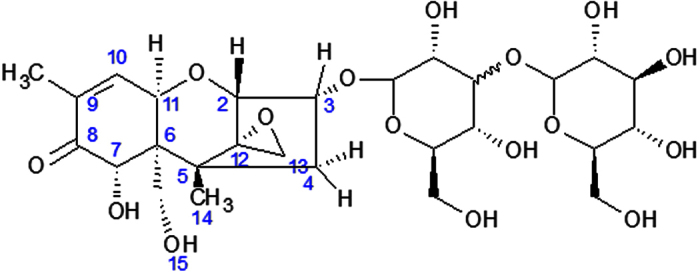
Proposed chemical structure of deoxynivalenol-diglucoside.

Using a non-targeted high resolution (HR)-MS^E^ approach in the two aphid species fed with DON (100 mg l^−1^), we were able to recover DON-diglucosides, for which unfortunately reference standards are not available. Other possible conjugates such as DON-GSH (glutathiones) which are known to be produced in other organisms as type II biotransformation products were investigated, however, we were not able to detect DON-GSH in any of the aphid samples confirming the GST enzyme tests. The DON-fed aphid samples were analyzed and discrepancies between aphid species were checked. Three peaks at retention times of 3.98 min, 4.25 min and 4.42 min were observed, corresponding to different structural isomers of DON-diglucoside. For these specific retention times, the measured and theoretical masses were investigated via MassLynx^TM^ software-analysis. The obtained molecular formula was C_27_H_40_O_16_Na^+^ with a mass of 643.2214 (theoretical) and 643.2202 (measured), resulting in a mass error of −1.9 ppm. The chemical structure of this diglucoside is proposed in Fig. 4. In addition, in view of the presence of DON-3G, we hypothesize here that the presence of the diglucoside form results from a sequential process in which the conversion of DON to DON-3G is the primary step. In order to understand the insertion of the glucose molecules of the three structural isomers, a study via nuclear magnetic resonance (NMR) is interesting. NMR can provide a conclusive structure-elucidation of the complete molecule including spins, alfa- and beta-isomerization, etc. However, it would be limited due to the extremely small amounts that can be isolated from aphid samples.

## Discussion

The trichothecene DON is a sesquiterpenoid mycotoxin produced by several *Fusarium* species and is toxic for most eukaryotic cells. In the present study, we assessed the toxicity of DON for two aphid species: the English grain aphid *S. avenae* and the pea aphid *A. pisum*. Dietary exposure of both aphids to DON showed that *S. avenae* were tolerant to DON compared to *A. pisum*. To explain this unique difference in toxicity of DON between the two aphid species three hypotheses were tested.

First, we examined the amino acid sequence of the gene encoding RPL3, the target molecule of DON, in both aphid species. Trichodermin and other sesquiterpenoids of the same group are known inhibitors of the peptidyltransferase center of eukaryotic ribosomes, and thereby block protein synthesis^23^,^24^. Research showed that RPL3 plays an essential role in the formation of this peptidyltransferase center^10^,^25^,^26^. One of the resistance mechanisms to DON identified in yeast is the modification of this ribosomal target by amino acid changes in RPL3^27^. Mitterbauer *et al*.^13^ used yeast as a model system to identify several mutations in the gene encoding RPL3 (*e.g.* W255C, a change of tryptophan into cysteine at position 255), which confer resistance to trichothecenes, in particular to DON. However, the amino acid sequence of *S. avenae*’s RPL3 showed none of these mutations. In addition, no functional aberrations were observed between the amino acid sequence of RPL3 from *S. avenae* and the predicted RPL3 from *A. pisum*. Amino acids at places 190 and 382 are valine (V) for *A. pisum*, but isoleucine (I) for *S. avenae*. However, when comparing the chemical structure of these two amino acids we concluded that this cannot explain the improved survival of *S. avenae* in presence of DON compared to *A. pisum*. Not the whole amino acid sequence of the *S. avenae* RPL3 was picked up, leaving seven amino acids undetermined at the end. As our sequencing data of the RPL3 of *S. avenae* did not show the mutations reported in yeast, we can conclude that the target of DON, the gene encoding RPL3, is not the reason of the tolerance in grain aphids.

Second, we investigated the hypothesis of Mitterbauer *et al*.^13^ stating that organisms might be heterozygous for the RPL3 locus. In this hypothesis, native ribosomes originating from one allele could be preferentially dismantled and degraded *in vivo* upon DON exposure. In consequence, the remaining fraction of resistant ribosomes on the second allele could allow the synthesis of new ribosomal proteins, eventually leading to a higher and steady-state level of modified RPL3 protein in the ribosomes. This hypothesis was validated by these researchers^27^ via an engineered tomato RPL3 containing the mutations of the yeast RPL3, which resulted in an adaptation but not in a constitutive tolerance against DON pointing to the semi-dominant nature of this tolerance. Expression of this aberrant gene in tobacco showed that the aberrant RPL3 protein was not utilized when the wild-type RPL3 protein was present, unless the transgenic plants were challenged with sub-lethal amounts of DON. Indeed, after toxin treatment in a dose-dependent manner, they noticed an accumulation of the modified protein due to the selection pressure in the presence of DON^13^. We investigated this hypothesis with our two aphid species in an experimental setup where we fed the aphids with increasing DON-concentrations over a period of 2 weeks. However, we did not detect any augmented tolerance especially in *A. pisum* when gradually exposed to increasing DON doses.

Finally, we investigated whether DON was subject to a type II biotransformation process in aphids. Metabolisation of the trichothecene monoacetoxyscirpenol in insects has been reported once before in literature although the type of metabolisation nor the detoxification was addressed in detail at that time^28^,^29^. In general, detoxification of xenobiotics is well-known in insects. Some major groups of genes encoding metabolic enzymes to detoxify xenobiotics like insecticides and plant derived metabolites have been described i.e., glucosyltransferases esterases, P450s, and GSTs^30^,^31^,^32^.

We were able to demonstrate the presence of DON-3G in both aphid species pointing to a glucosyltransferase involved in the detoxification of DON in aphids. Moreover, the tolerant *S. avenae* species converted DON to DON-3G more efficiently than the susceptible *A. pisum*. To our knowledge, this is the first time that the conversion of DON to DON-3G is reported in animal species. To date, DON glucosylation has solely been reported in plant cells. In plants, a vast number of genes that code for putative UDP-glycosyltransferases (UGTs) has been revealed^19^. In barley and *Brachypodium distachyon,* genes from the UGT family with potential relevance for DON tolerance have functionally been characterized^33^,^34^. Although DON glucosylation has never been reported in animals, genes encoding for UGTs are known to be present in insects. They catalyze the conjugation of a range of diverse small lipophilic compounds with polar compounds (*i.e.* carbohydrates) to produce glucosides, and as such they play an important role in type II detoxification processes of xenobiotics in insects^35^. However, the presence of these UGTs have never been linked with mycotoxin glucosylation. It has been shown that the insect UGT enzymes typically use UDP-glucose as the main sugar donor unlike vertebrate UGTs which mainly utilize UDP-glucuronic acid^36^. In aphids, up-regulated ecdysteroid UDP-glucosyltransferases have been associated with tolerance of cotton aphids to the neonicotinoid insecticide thiamethoxam. In addition, they have been shown to be expressed upon migration of aphids from primary to secondary hosts^37^. These examples illustrate the plethora of biological processes in which UGT enzymes are involved.

Although we provide valuable evidence for a role of glucosylation in DON-detoxification in aphids, several other detoxification enzymes have been described in aphids for coping with xenobiotics (*e.g.* secondary compounds from plants or insecticides); including P450s, GSTs, esterases and oxidoreductases^38^,^39^,^40^,^41^,^42^,^43^. Some of these enzymes are known to be involved in the detoxification of mycotoxins^20^. In honeybees *Apis mellifera*, the larvae of *Trichoplusia ni* and corn earworms *Helicoverpa zea*, it has been demonstrated that P450s play a role in bio-activation or detoxification of aflatoxin B1 produced by *Aspergillus* spp^44^,^45^. However, in this study we showed that GSTs and P450s were not involved.

Using a non-targeted HR-MS^E^ approach, we were able to detect DON-diglucosides (via HR-MS^E^) in both *S. avenae* and *A. pisum* aphids. To date, the only report on the presence of DON-diglucosides was in beer, where oligoglucosylated DONs with up to four bound hexose units were present^46^. Remarkably, although detoxification of DON through conjugation with glutathione (DON-GSH) has been observed in plants^20^,^21^ and in many animal species^15^ no glutathione derivatives of DON were observed during the HR-MS^E^ analyses of our aphid samples.

The question remains why *S. avenae* is able to convert DON to DON-3G more efficiently than *A. pisum*. Insights might come from the knowledge that *S. avenae* occurs on cereal ears which are often colonized by *Fusarium* spp. producing DON, while *A. pisum* occurs on plant species that are no hosts for DON producing *Fusarium* spp^47^. It is remarkable that *A. pisum* although it disposes of a very large arsenal of UGTs compared to other insects^35^, does not convert DON efficiently to DON-3G which points to the substrate specificity of these enzyme. Consequently, we might speculate on adaption by co-evolution in *S. avenae*. It has been reported before that insects are capable to develop tolerance when exposed to a toxin over many generations. *Drosophila melanogaster* larvae which were exposed to *Aspergillus nidulans* over 26 generations displayed higher survival rates in the presence of *A. nidulans* and a higher tolerance to the mycotoxin sterigmatocytin (*i.e.* an aflatoxin precursor) compared to control lines^48^.

Finally, it is tempting to argue on the origin of the glucosyltransferase in aphids. Although we do not provide evidence in the present study, one of the possibilities of acquiring this specific glucosyltransferase is through horizontal gene transfer. Horizontal transfer of a bacterial gene encoding an enzyme which detoxifies the plant toxin hydrogen cyanide to plant-feeding spider mites (*Tetranychus urticae*) has previously been shown in a model system using *Phaseolus lunatus*. This event resulted in an increased survival upon exposure of the insects to hydrogen cyanide^49^. In our model system using aphids, a potential source of bacterial enzymes degrading the mycotoxin DON are endosymbionts. Aphids are insects known to contain many obligatory (e.g. *Buchnera* aphidicola) and facultative symbionts^50^. The latter group is mutualistic in the context of various ecological interactions. Although it has been suggested that they are involved in detoxification of various toxic metabolites, solid evidence for this feature is still lacking. Due to this, the direct or indirect (e.g. though horizontal gene transfer) role of symbionts in detoxification processes remains enigmatic^50^,^51^. Nevertheless, this discussion illustrates a possible new aspect of the chemical warfare between plants and insects.

## Methods

### Insects and chemicals

Laboratory stock cultures of cereal aphids *S. avenae* and pea aphids *A. pisum* were maintained on wheat seedlings and young broad bean plants, respectively, under standard conditions of 22 °C and a photoperiod of 16 h light with a relative humidity ranging from 50% to 70%, stimulating parthenogenesis^52^.

DON was kindly provided by M. Lemmens (BOKU, Vienna, Austria). Purity of the provided stock standard was >99%. A stock solution was prepared by dissolving 5 mg DON in 5 ml (1 mg ml^−1^) sterile water and stored at −20 °C. DON-3G (50 μg ml^−1^) was purchased at Sigma-Aldrich (Diegem, Belgium).

### Survival of *S. avenae* and *A. pisum* when feeding from DON and DON-3G

To determine the effect of DON and DON-3G on the survival of *S. avenae* and *A. pisum*, DON and DON-3G were added to the artificial aphid diet based on formulation A from Prosser and Douglas^53^ to a final concentration of 0.5, 1 or 3 and 100 mg l^−1^. Sterile water was added to the artificial diet as control (0 mg l^−1^ DON or DON-3G). For both aphid species, there were three aphid feeding apparatuses prepared as described by Sadeghi, *et al*.^54^ for all treatments and control. Each apparatus contained ten randomly picked nymphs who could feed on a parafilm sachet containing 200 μl of the diet. Over a period of three days the surviving nymphs were counted. Abbott’s formula^55^ was used to correct the survival rates: (nTa/nCa)* 100, nTa represents the number of survivors after treatment and nCa the number of survivors in the control treatment. Statistical differences (P < 0.05) between aphid survival when feeding from different DON concentrations were analyzed by using non-parametric Kruskal-Wallis analysis followed by a Dunn’s test to perform pairwise comparisons using IBM SPSS (IBM Corp. Released 2013. IBM SPSS Statistics for Windows, Version 22.0. Armonk, NY: IBM Corp.). These experiments were repeated at least two times with 4 samples per treatment.

To determine the long-term effect of DON on the survival of *S. avenae* and *A. pisum,* aphids were gradually exposed to increasing concentrations: 0 → 0.5 → 1 → 3 → 5 mg l^−1^ DON in aphid feeding apparatus^54^. For both aphid species, there were six aphid feeding apparatus prepared for the treatment and for the control (0 mg l^−1^ DON). Each apparatus contained five nymphs produced by adult aphids within 24 h (day 0). In these experiments, the survival of the nymphs was checked once every day and the diet was changed every two days. Statistical different survival percentages (P < 0.05) between treatments at different time points in the long-term survival experiment were analyzed using one-sided t-tests (SPSS Statistics 22).

For non-targeted and targeted LC-MS/MS analyses *S. avenae* and *A. pisum* aphids (in different developmental stages) were taken from the laboratory stock cultures and put in aphid feeding apparatuses containing 0 or 100 mg l^−1^ DON. After 40 h the surviving aphids were stored at −20 °C until analysis. For each aphid species we performed five replications of aphids that were fed with 0 and 100 mg l^−1^ DON. In addition, after 40 h of feeding the artificial diet of all repeats were analyzed.

### Analysis of the ribosomal protein L3

RNA from *S. avenae* and *A. pisum* aphids was extracted using TRI reagent (Sigma-Aldrich) according to the manufacturer’s instructions. The extracted RNA was quantified using a Quantus Fluorometer (Promega, Madison, WI, USA). With a GoScript Reverse Transcription System (Promega), first-strand cDNA was synthesized. The PCR reactions were performed in a total reaction volume of 25 μl, consisting of 0.125 μl GoTaq DNA polymerase (Promega), 5 μl of 5× GoTaq buffer colorless (Promega), 1.25 μl dNTPs, 1 μl of each primer (5 μM), 14.625 μl nuclease-free water (Promega) and 2 μl of the cDNA. The RPL3 sequence was picked up in two parts (p1 and p2) using following primers (5′-3′): p1_F GCACATCCACTTTCGTCAAG, p1_R CTAGGATGCCATGCTCCAAT, p2_F ACCAAGGGTCGTGGATACAA and p2_R CGCTGTGGCTTTCTCTTCTT. PCR analysis was performed with a Bio-Rad T100 Thermal Cycler, the thermocycler profile used was: 5 min at 95 °C followed by 35 cycles at 95 °C for 30 s, 59.7 °C (p1) or 60 °C (p2) for 20 s and 72 °C for 60 s, followed by 72 °C for 10 min and cooled down to 15 °C. The remaining product was purified using the E.Z.N.A. Cycle-Pure Spin kit (VWR, Leuven, Belgium) and send to LGC Genomics (Berlin, Germany) for single sample DNA sequencing. Sequence alignment between *S. avenae* and *A. pisum* was done with ClustalW Multiple alignment in Bioedit.

### Sample preparation and targeted LC-MS/MS analysis

Individual mycotoxin solid standards (1 mg) of DON, 3-ADON, 15-ADON and DOM-1 (internal standard) were purchased from Sigma-Aldrich NV/SA (Bornem, Belgium). DON-3G (50.2 ng μl^−1^ in acetonitrile) was obtained from Biopure Referenzsubstanzen GmbH (Tulln, Austria). All mycotoxin solid standards were dissolved in methanol (1 mg ml^−1^), and were stored at −18 °C. Working solutions of DON, 3-ADON, 15-ADON and DOM (10 ng μl^−1^) were prepared in methanol and stored at −18 °C, while DON-3G (50.2 ng μl^−1^) was dissolved in acetonitrile and stored at 4 °C. The targeted LC-MS/MS analysis was performed using a Waters Acquity UPLC system coupled to a Quattro Premier XE mass spectrometer (Waters, Milford, MA, USA) equipped with an electrospray interface in positive mode (ESI^+^). Following MRM-traces were monitored: DON (297 > 203.3; 249.4), 3-ADON (339.2 > 231.2; 203.1), 15-ADON (339.1 > 137.1; 321.2) and DON-3G (476.1>248.6 ; 296.9). LC-MS/MS parameters are described in detail by De Boevre, *et al*.^56^. MassLynx^TM^ version 4.1. and QuanLynx^®^ version 4.1. software (Waters, Milford, MA, USA) were used for data acquisition and processing.

Aphid samples were collected, crushed and individually weighed in recipients. According to their weight, 500 ng g^−1^ of DOM internal standard (10 ng μl^−1^) was added. A matrix-matched calibration curve with a linear range of 0 ng g^−1^ to 1500 ng g^−1^ for DON, 3-ADON, 15-ADON and DON-3G with non-contaminated *S. avenae* aphids was prepared. The reference standards were allowed to equilibrate for 15 min. An extraction with 1.5 ml acetonitrile/water/acetic acid (79/20/1, v/v/v) was performed, and the samples were vigorously vortexed for 1 min. The sample extract was centrifuged at 4,307 g for 1 min and the supernatant was collected in a small test tube using a glass Pasteur pipette with a bulb. This process was repeated twice. The organic mycotoxin-mixture was evaporated until dry under N_2_ at 60 °C using the TurboVap^®^ LV (Biotage, Dusseldorf, Germany), and redissolved in 150 μl of injection solvent (50/50 v/v, H_2_O/MeOH (95/5, v/v), 0.1% of HCOOH + 10 mM of HCOONH_4_ [solvent A]; MeOH/H_2_O (95/5, v/v), 0.1% of HCOOH + 10 mM of HCOONH_4_ [solvent B]). Finally, the redissolved sample was vortexed for 3 minutes, collected in an Ultrafree-MC centrifugal device (0.22 μm, Millipore, Bedford, MA, USA) and centrifuged for 10 minutes at 10,000 g.

To confirm the presence of DON, 3-ADON, 15-ADON and DON-3G, two transitions between precursor and fragments were monitored. According to the Commission Decision of the Council Directive 96/23/EC in August 12, 2002 concerning the performance of analytical methods and the interpretation of results (2002/657/EC, 2002), a system of identification points was applied to interpret the data^57^. The first criterion indicates that the relative retention time, relative to the internal standard DOM-1, should not exceed 2.5%. The second identification point involved that the relative abundance of both transitions should not exceed the range of 20% to 50%, depending on the relative intensity between the transitions. Also, all MRM-transitions should possess a signal-to-noise (s/n) ratio higher than 3:1 (2002/657/EC, 2002).

Statistical differences (P < 0.05) between concentrations of DON, DON-3G and total titer retrieved in aphids were analyzed by using a one-way ANOVA with a post-hoc Tukey test (SPSS Statistics 22).

### Sample preparation and non-targeted LC-MS^E^ analysis

To an exact amount of the aphid sample (individually checked), 750 μl of extraction solvent acetonitrile/water/acetic acid (79/20/1, v/v/v) was added. Using a glass spatula, the aphids samples were crushed until a homogeneous mass was obtained. The spatula was rinsed with 750 μl of extraction solvent. The organic mixture was vigorously vortexed for 1 minute, followed by centrifugation at 4,307 g for 1 minute. The obtained supernatant was transferred into a small test tube. To extract the maximum amount of mycotoxins, 1.5 ml of extraction solvent was added to the centrifuged residue. The vortex and centrifugation step were repeated, and the remaining supernatant was transferred into the same test tube. The organic mycotoxin-mixture was evaporated until dryness under N_2_ at 60 °C using the TurboVap^®^ LV (Biotage, Dusseldorf, Germany). The residue was redissolved with 150 μl of MeOH/CAN/H_2_O (30/30/40, v/v/v) and centrifuged in an Ultrafree^®^-MC centrifugal device (0.22 μm) for 5 minutes at 14,000 g.

DON and its derivatives (DON-3G, 3-ADON, 15-ADON, DON-GSH, DON-diglucosides, DON-triglucosides and DON-tetraglucosides) were investigated using UPLC/Q-TOF-MS with the MS^E^ data acquisition strategy. The LC instrument used was an Acquity UPLC^TM^ system (Waters Milford, MA, USA) with a ZORBAX RRHD Eclipse Plus C18 (1.8 μm, 2.1 × 100 mm) from Agilent Technologies (Diegem, Belgium). The mobile phase consisted of H_2_O/MeOH (95/5, v/v) containing 0.1% of HCOOH and 10 mM of HCOONH_4_ [solvent A] and MeOH/H_2_O (95/5, v/v) containing 0.1% of HCOOH and 10 mM of HCOONH_4_ [solvent B]. The following gradient elution program was applied: 0–0.5 min: 0% B, 0.5–20 min: 0–99% B, 20–21 min: 99% B, 21–24 min: 0% B, 24–28 min: 0% B. The flow rate was 0.4 ml min^−1^. The column temperature was set at 30 °C, and the temperature of the autosampler was 10 °C. Five μl of the sample was injected. Instrument control and data processing were carried out by MassLynx^TM^ version 4.1. software (Waters, Milford, MA, USA). The Q-TOF MS instrument used was a Synapt G2-Si MS system (Waters, Milford, MA, USA). The data acquisition mode was TOF MS^E^ in ESI^+^ mode. The data acquisition ranged from 50 Da to 1200 Da with a 0.1 s scan time. The MS source temperature was set at 150 °C, and the desolvation temperature was set at 500 °C with a desolvation gas flow set at 800 l h^−1^ and a cone gas flow at 100 l h^−1^. The capillary voltage was 2.8 kV and the sampling cone voltage was 30 V. The collision energy was set as 45 eV–60 eV ramp (trap) for the high-energy scan. Data was collected in continuum mode and the mass was corrected to ensure accuracy during the MS analysis after acquisition using leucine enkephaline (200 pg μl^−1^) at a flow rate of 100 μl min^−1^ as lock mass compound. HRMS data were processed using MassLynx^TM^ software and compounds were identified after applying lockspray correction, extracting the chromatogram and generating the molecular formula from the exact mass.

## Additional Information

**How to cite this article**: Zutter, N. D. *et al*. Aphids transform and detoxify the mycotoxin deoxynivalenol via a type II biotransformation mechanism yet unknown in animals. *Sci. Rep.* **6**, 38640; doi: 10.1038/srep38640 (2016).

**Publisher's note:** Springer Nature remains neutral with regard to jurisdictional claims in published maps and institutional affiliations.

## Supplementary Material

Supplementary Figure 1 and 2

## Figures and Tables

**Figure 1:**
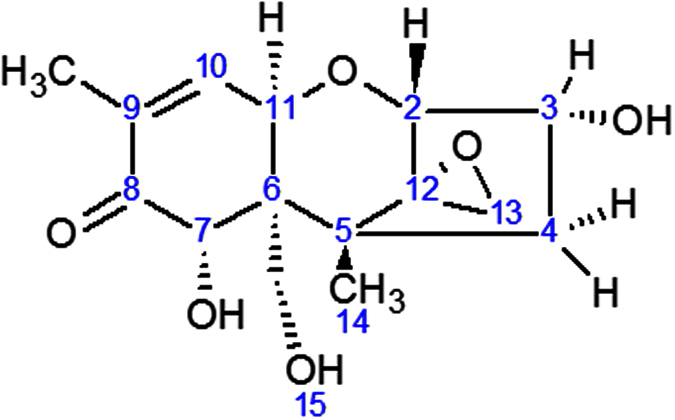
Chemical structure of the mycotoxin deoxynivalenol (DON).

**Table 1:** Percentage survival ± S.E. of *S. avenae* aphids and *A. pisum* aphids feeding from diet containing different concentrations of deoxynivalenol.

	Concentration	*Sitobion avenae*	*Acyrthosiphon pisum*
Control		97 ± 3a	100 ± 0a
DON	0.5 mg l^−1^	93 ± 3a (p = 1.000)	62 ± 6bc (p = 0.024)
	1 mg l^−1^	93 ± 2a (p = 1.000)	48 ± 9c (p = 0.006)
	3 mg l^−1^	93 ± 4a (p = 1.000)	50 ± 11c (p = 0.012)
DON-3G	0.5 mg l^−1^	88 ± 6a (p = 1.000)	88 ± 5ab (p = 1.000)
	1 mg l^−1^	89 ± 5a (p = 1.000)	96 ± 2ab (p = 1.000)
	3 mg l^−1^	81 ± 7a (p = 0.160)	88 ± 6ab (p = 1.000)

Each treatment consisted of 3 feeding apparatuses each containing 10 nymphs per species. Different letters indicate significant differences (P < 0.05) between treatments using a two-sided non-parametric Kruskall-Wallis test followed by a Dunn’s test for pairwise comparisons. The statistical significances in pairwise comparisons with the control treatments are depicted as a p-value between brackets (this p-value was corrected for multiple pairwise comparisons).
