# Supplementary material for: Aphids transform and detoxify the mycotoxin deoxynivalenol via a type II biotransformation mechanism yet unknown in animals
Source: Sci Rep. 2016 Dec 8;6:38640. doi: 10.1038/srep38640 (PMC5144147; doi:10.1038/srep38640)

Aphids transform and detoxify the mycotoxin deoxynivalenol via a type II biotransformation mechanism yet unknown in animals

N. De Zutter[1,2*], K. Audenaert[2*], N. Arroyo-Manzanares[3*], M. De Boevre[3], C. Van Poucke[3], S. De Saeger[3], G. Haesaert[2*] and G. Smagghe[1*]

[1] Department of Crop Protection, Laboratory of Agrozoology, Faculty of Bioscience Engineering, Ghent University, Coupure Links 653, 9000 Ghent, Belgium; [2] Department of Applied Biosciences, Faculty of Bioscience Engineering, Ghent University, Valentin Vaerwyckweg 1, 9000 Ghent, Belgium; [3] Department of Bioanalysis, Laboratory of Food Analysis, Ghent University, Ottergemsesteenweg 460, 9000 Ghent, Belgium

Correspondence: kris.audenaert@ugent.be

Supplementary Figure 1 Alignment of ribosomal protein L3 (RPL3) sequences from different aphid species and yeast. RPL3 sequences of *Sitobion avenae*, predicted 60S RPL3 *Acyrthosiphon pisum* (NCBI Reference Sequence: XP_001951042.1), predicted 60S RPL3 *Diuraphis noxia* (NCBI Reference Sequence: XP_015366271.1), 60S RPL3 *Aphis gossypii* (GenBank: AGT79995.1) and RPL3 *Saccharomyces cerevisiae* (GenBank: AAA88732.1) were aligned using BioEdit Sequence Alignment Editor (ClustalW Multiple alignment). Amino acid changes (S2P, P9L, W255R, W255C, H256Y) identified from *S. cerevisiae* conferring deoxynivalenol resistance are given below the sequences

```
SaRPL3          MSHRKFSAPR HGSMGFYPKK RARRHRGRVK SFPKDDPSKP IHLTAFIAYK AGMTHVVREA DRPGSKLNKK :70
ApRPL3          MSHRKFSAPR HGSMGFYPKK RARRHRGRVK SFPKDDPSKP IHLTAFIAYK AGMTHVVREA DRPGSKLNKK
DnRPL3          MSHRKFSAPR HGSMGFYPKK RARRHRGRVK SFPKDDPSKP IHLTAFIAYK AGMTHVVREA DRPGSKLNKK
AgRPL3          MSHRKFSAPR HGSMGFYPKK RARRHRGRVK SFPKDDPSKP IHLTAFIAYK AGMTHVVREA DRPGSKLNKK
ScRPL3          MSHRKYEAPR HGHLGFLPRK RAASIRARVK AFPKDDRSKP VALTSFLGYK AGMTTIVRDL DRPGSKFHKR
                 P      L

SaRPL3          EIVEPVTILE APPMIIVGVV GYVETPYGLK PLKTVFAEHL SEDCRRRFYK NWYKSKKKAF VKYSRKWQDE :140
ApRPL3          EIVEPVTILE APPMIIVGVV GYVETPYGLK PLKTVFAEHL SEDCRRRFYK NWYKSKKKAF VKYSRKWQDE
DnRPL3          EIVEPVTILE APPMIIVGVV GYVETXYGLK PLKTVFAEHL SEDCRRRFYK NWYKSKKKAF VKYSRKWQDE
AgRPL3          EIVEPVTILE APPMIIVGVV GYVETPYGLK PLKTVFAEHL SEDCRRRFYK NWYKSKKKAF VKYSRKWQDE
ScRPL3          EVVEAVTVVD TPPVVVVGVV GYVETPRGLR SLTTVWAEHL SDEVKRRFYK NWYKSKKKAF TKYSAKYAQD

SaRPL3          NGKRQIAKDL GKIAKYSKVI RVVAHTQMKL LKKRQKKAHI MEIQVNGGTI AEKVQWAKEH FEKPVPVSHV :210
ApRPL3          NGKRQIAKDL GKIAKYSKVI RVVAHTQMKL LKKRQKKAHI MEIQVNGGTV AEKVQWAKEH FEKPVPVSHV
DnRPL3          NGKRQIAKDL GKIAKYSKVI RVIAHTQMKL LKKRQKKAHI MEIQVNGGTV AEKVQWAKEH FEKPVPVSHV
AgRPL3          NGKRQIAKDL GKIAKYSKVI RVVAHTQMKL LKKRQKKAHI MEIQVNGGTV AEKVQWAKEH FEKPVPVSHV
ScRPL3          GAG--IEREL ARIKKYASVV RVLVHTQIRK TPLAQKKAHL AEIQLNGGSI SEKVDWAREH FEKTVAVDSV

SaRPL3          FAPDEMIDCI GVTKGRGYKG VTSRWHTKKL PRKTHKGLRK VACIGAWHPS RVQFTVARAG QKGYHHRTEI :280
ApRPL3          FAPDEMIDCI GVTKGRGYKG VTSRWHTKKL PRKTHKGLRK VACIGAWHPS RVQFTVARAG QKGYHHRTEI
DnRPL3          FAPDEMIDCI GVTKGRGYKG VTSRWHTKKL PRKTHKGLRK VACIGAWHPS RVQFTVARAG QKGYHHRTEI
AgRPL3          FAPDEMIDCI GVTKGRGYKG VTSRWHTKKL PRKTHKGLRK VACIGAWHPS RVQFTVARAG QKGYHHRTEI
ScRPL3          FEQNEMIDAI AVTKGHGFEG VTHRWGTKKL PRKTHRGLRK VACIGACHPA HVMWSVARAG QRGYHSRTSI
                                                                  RY
                                                                  C

SaRPL3          NKKIYRIGLG IHTKDGKVIK NNASTEYDLT EKTITPMGGF PHYGEVNNDF LMIKGCCVGP KKRVITLRKS :350
ApRPL3          NKKIYRIGLG IHTKDGKVIK NNASTEYDLT EKTITPMGGF PHYGEVNNDF LMIKGCCVGP KKRVITLRKS
DnRPL3          NKKIYRIGLG IHTKDGKVIK NNASTEYDLT EKTITPMGGF PHYGEVNNDF LMIKGCCVGP KKRVITLRKS
AgRPL3          NKKIYRIGLG IHTKDGKVIK NNASTEYDLT EKTITPMGGF PHYGEVNNDF LMIKGCCVGP KKRVITLRKS
ScRPL3          NHKIYRVGKG DDEANG---- ---ATSFDRT KKTITPMGGF VHYGEIKNDF IMVKGCIPGN RKRIVTLRKS

SaRPL3          LLVHTKRAAL ESINLKFIDT SSKFGHGRFQ TIADKAAFMG PLKKDRIREE E------- :410
ApRPL3          LLVHTKRAAL ESINLKFIDT SSKFGHGRFQ TVADKAAFMG PLKKDRIREE EKATAAAK
DnRPL3          LLVHTKRAAL ESINLKFIDT SSKFGHGRFQ TVADKAAFMG PLKKDRIREE EKATAAAK
AgRPL3          LLVHTKRAAL ESINLKFIDT SSKFGHGRFQ TVADKAAFMG PLKKDRIREE EKATAAAK
ScRPL3          LYTNTSRKAL EEVSLKWIDT ASKFGKGRFQ TPAEKHAFMG TLKKDL---- --------
```

Supplementary Figure 2 Chemical structure of the DON derivatives DON-3G (A), 3-ADON (B) and 15-ADON (C)

A

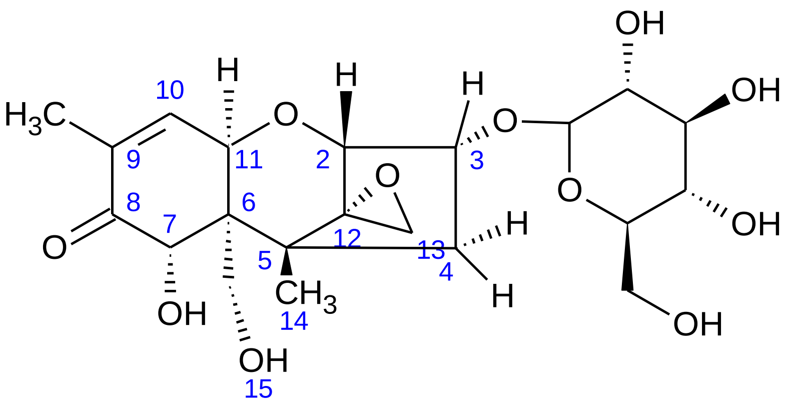

B

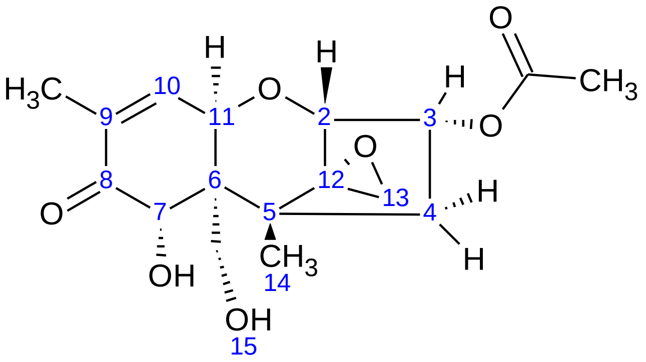

C

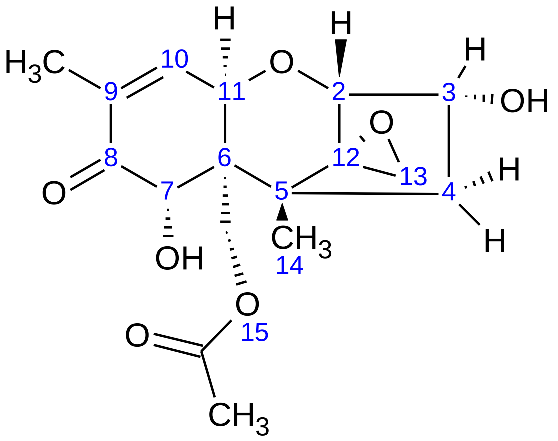

Supplement: Supplementary Figure 1 and 2 [file srep38640-s1.pdf]
